# Climate change considerations are fundamental to management of deep‐sea resource extraction

**DOI:** 10.1111/gcb.15223

**Published:** 2020-07-06

**Authors:** Lisa A. Levin, Chih‐Lin Wei, Daniel C. Dunn, Diva J. Amon, Oliver S. Ashford, William W. L. Cheung, Ana Colaço, Carlos Dominguez‐Carrió, Elva G. Escobar, Harriet R. Harden‐Davies, Jeffrey C. Drazen, Khaira Ismail, Daniel O. B. Jones, David E. Johnson, Jennifer T. Le, Franck Lejzerowicz, Satoshi Mitarai, Telmo Morato, Sandor Mulsow, Paul V. R. Snelgrove, Andrew K. Sweetman, Moriaki Yasuhara

**Affiliations:** ^1^ Integrative Oceanography Division and Center for Marine Biodiversity and Conservation Scripps Institution of Oceanography University of California, San Diego La Jolla CA USA; ^2^ Institute of Oceanography National Taiwan University Taipei Taiwan; ^3^ School of Earth and Environmental Sciences University of Queensland St Lucia Qld Australia; ^4^ Life Sciences Department Natural History Museum London UK; ^5^ Institute for the Oceans and Fisheries The University of British Columbia Vancouver BC Canada; ^6^ IMAR Instituto do Mar, and Instituto de Investigação em Ciências do Mar – Okeanos da Universidade dos Açores Horta Portugal; ^7^ Instituto de Ciencias del Mar y Limnología Universidad Nacional Autónoma de México Mexico City Mexico; ^8^ Australian National Centre for Ocean Resources and Security University of Wollongong Wollongong NSW Australia; ^9^ Department of Oceanography University of Hawaii at Manoa Honolulu HI USA; ^10^ Faculty of Science and Marine Environment Universiti Malaysia Terengganu Kuala Terengganu Malaysia; ^11^ Ocean Biogeochemistry and Ecosystems Group National Oceanography Centre Southampton UK; ^12^ Global Ocean Biodiversity Initiative Seascape Consultants Ltd. Romsey UK; ^13^ Jacobs School of Engineering University of California San Diego La Jolla CA USA; ^14^ Marine Biophysics Unit Okinawa Institute of Science and Technology Graduate University Okinawa Japan; ^15^ Instituto Ciencias Marinas y Limnológicas Universidad Austral de Chile Valdivia Chile; ^16^ Department of Ocean Sciences and Biology Department Memorial University of Newfoundland St. John's NL Canada; ^17^ The Lyell Centre for Earth and Marine Science and Technology Heriot Watt University Edinburgh UK; ^18^ School of Biological Sciences and Swire Institute of Marine Science The University of Hong Kong Hong Kong SAR China

**Keywords:** biodiversity maintenance, bottom fishing, climate projections, conservation, deep ocean, deep‐seabed mining, environmental management, habitat suitability modeling, larval connectivity modeling

## Abstract

Climate change manifestation in the ocean, through warming, oxygen loss, increasing acidification, and changing particulate organic carbon flux (one metric of altered food supply), is projected to affect most deep‐ocean ecosystems concomitantly with increasing direct human disturbance. Climate drivers will alter deep‐sea biodiversity and associated ecosystem services, and may interact with disturbance from resource extraction activities or even climate geoengineering. We suggest that to ensure the effective management of increasing use of the deep ocean (e.g., for bottom fishing, oil and gas extraction, and deep‐seabed mining), environmental management and developing regulations must consider climate change. Strategic planning, impact assessment and monitoring, spatial management, application of the precautionary approach, and full‐cost accounting of extraction activities should embrace climate consciousness. Coupled climate and biological modeling approaches applied in the water and on the seafloor can help accomplish this goal. For example, Earth‐System Model projections of climate‐change parameters at the seafloor reveal heterogeneity in projected climate hazard and time of emergence (beyond natural variability) in regions targeted for deep‐seabed mining. Models that combine climate‐induced changes in ocean circulation with particle tracking predict altered transport of early life stages (larvae) under climate change. Habitat suitability models can help assess the consequences of altered larval dispersal, predict climate refugia, and identify vulnerable regions for multiple species under climate change. Engaging the deep observing community can support the necessary data provisioning to mainstream climate into the development of environmental management plans. To illustrate this approach, we focus on deep‐seabed mining and the International Seabed Authority, whose mandates include regulation of all mineral‐related activities in international waters and protecting the marine environment from the harmful effects of mining. However, achieving deep‐ocean sustainability under the UN Sustainable Development Goals will require integration of climate consideration across all policy sectors.

## Graphical abstract

As use of the deep sea and its resources intensifies, consideration of climate change should become integral to planning and management. A suite of climate and biological modeling tools can inform holistic environmental management and decision making for the deep ocean on multiple fronts.

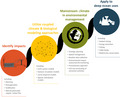

## 1 INTRODUCTION

Pervasive climate‐change impacts in the ocean, recognized as warming, acidification, deoxygenation, and a myriad of physical changes in water mass properties, affect all marine ecosystems, including those in the deep sea (Bindoff et al., 2019; Jones et al., 2014; Sweetman et al., 2017; Yasuhara & Danovaro, 2016). The deep ocean hosts a spectrum of living and non‐living resources that are increasingly vulnerable to exploitation (Mengerink et al., 2014) and provide key services that range from fisheries production and novel genetic resources to climate regulation and space for communication cables (Thurber et al., 2014). The deep sea can also influence the surface and coastal ocean; reduction in deep‐ocean oxygenation, for example, can affect coastal fisheries and livelihoods (Grantham et al., 2004). Deep‐sea bottom trawl and longline fisheries have developed on continental margins and on seamounts (underwater volcanos) to approximately 2,500 m water depth (Clark et al., 2016). Oil and gas extraction has deepened and now routinely occurs on continental margins to 3,000 m (Merrie et al., 2014). Rising demand for and declining grades of rare metals on land has led the mining industry to turn to the deep sea, targeting the vast reserves of cobalt, manganese, copper, nickel, silver, or gold stored in polymetallic nodules on abyssal plains at depths of 3,000–6,500 m (e.g., in the Clarion Clipperton Zone [CCZ] of the E. Pacific Ocean), cobalt‐rich ferromanganese crusts on seamounts (e.g., in the NW Pacific Ocean) and massive polymetallic sulfides deposited at deep hot springs (hydrothermal vents; e.g., on the Northern Mid‐Atlantic Ridge [MAR] or Southwest Indian Ridge, Indian Ocean; Miller, Thompson, Johnston, & Santillo, 2018).

The UN Convention on the Law of the Sea (UNCLOS) designated seafloor mineral resources in the international seabed areas (“the Area”) as the “common heritage of mankind” (Art. 136), and created the International Seabed Authority (ISA) to both oversee the development of these resources while also ensuring protection of the marine environment from harmful effects that may arise from this development (Art. 145). The ISA reaffirmed its commitment to protect the marine environment and to sustainably develop deep‐seabed mineral extraction under SDG 14 (Lodge, Segerson, & Squires, 2017). Deep‐water oil and gas reserves occur largely within national jurisdictions and activities are managed principally by states (Cordes et al., 2016). Deep‐sea fisheries resources in international waters are addressed by UNCLOS and the UN Fish Stocks Agreement and are managed by Regional Fisheries Management Organizations (RFMOs) under the direction of the Food and Agriculture Organization.

Concurrently, extraction of living and non‐living resources and other intensifying uses (e.g., CO_2_ disposal or cable laying) accelerate deep‐ocean (below 200 m) industrialization, with associated governance and regulations under active development (De Santo et al., 2019; Mengerink et al., 2014; Merrie et al., 2014). The developing nature of these industries and governance structures offer opportunities for proactive rather than reactive response to climate change (FAO, 2019; Levin & Le Bris, 2015). However, natural variability and the consequences of other human activities complicate ongoing ocean environmental and ecosystem changes. Ocean managers and scientists increasingly recognize the need to incorporate climate‐change considerations into monitoring and management of ocean use (e.g., Cheung, Jones, Reygondeau, & Frölicher, 2018; Dunn, Van Dover, et al., 2018; FAO, 2019; Fulton et al., 2018; Queirós, Fernandes, Genevier, & Lynam, 2018; Queirós et al., 2016; Thresher, Guinotte, Matear, & Hobday, 2015), but management measures often arise in response to change, rather than as part of a precautionary approach, limiting efficiency and benefits.

Here, we discuss the reasons for and strategies to integrate climate change into the management of human use of deep‐sea ecosystems, drawing examples from deep‐seabed mining (DSM). The nascent industry of DSM targets mineral resources in international waters and within some national exclusive economic zones (e.g., in Japan or Norway). While commercial mining of deep‐seabed minerals has yet to occur anywhere, the ISA is currently developing exploitation protocols, regulations, and guidelines (the Mining Code) for “the Area.” This framework offers a unique opportunity to embed climate consciousness in advance of resource extraction, in order to minimize harm under future climate change and enhance ecosystem resilience. We discuss potential impacts of climate change on ecosystem recovery following human activities, as well as the potential feedbacks of extractive activities on ocean regulation of climate. We present applications of climate projections, larval dispersal models, and habitat suitability models as research tools whose output can guide regional environmental management planning, impact assessment, and adaptive management. Finally, we discuss actions for mainstreaming climate into management of human use of the deep seabed, and how these actions may be facilitated by knowledge transfer, deep‐ocean observing, and cross‐sectoral policy integration.

## 2 CLIMATE IS CHANGING THE DEEP SEA, INCLUDING AREAS TARGETED FOR RESOURCE EXTRACTION

**FIGURE 1:**
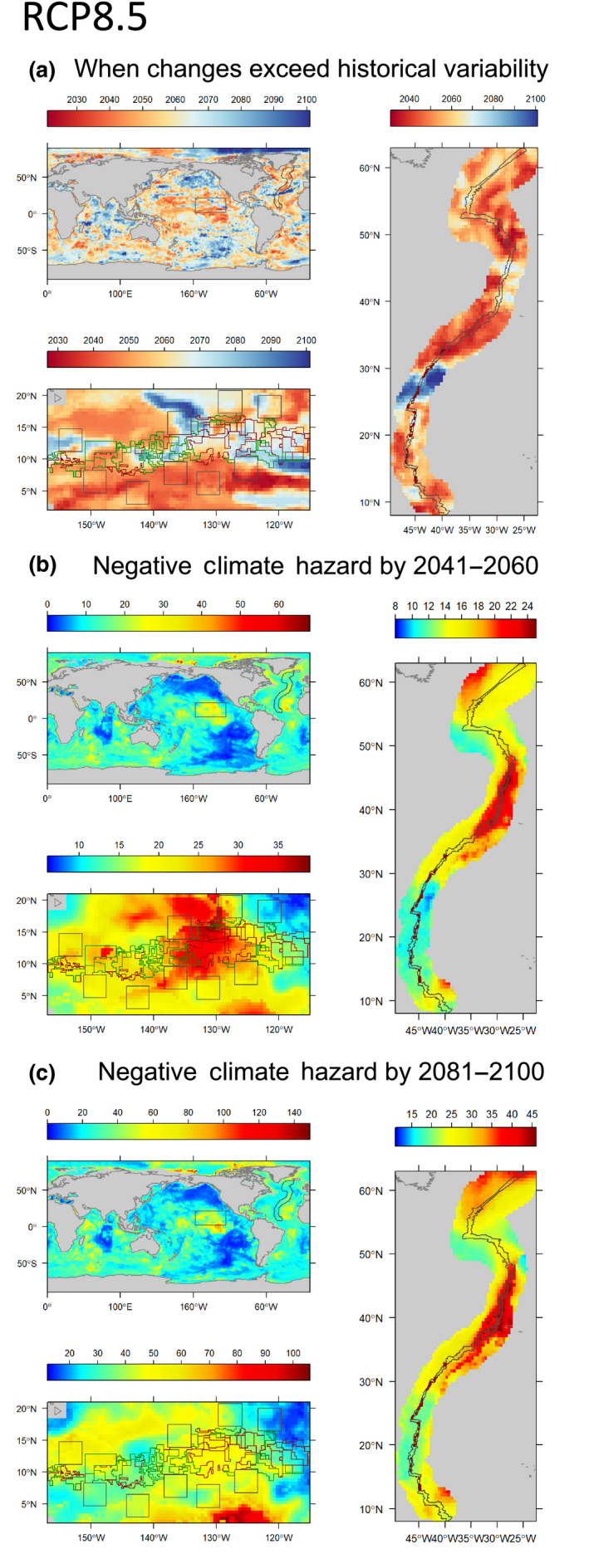
Climate projections for the RCP 8.5 scenario for the global seafloor and two regions targeted for deep‐seabed mining, the Clarion Clipperton Zone (CCZ; left panels) and the northern Mid‐Atlantic Ridge (MAR right panels). (a) Time of Emergence: the year when future variability exceeds historical variability for all climate changes in temperature, oxygen, pH, and food supply (i.e., annual standard deviation between 1951 and 2000). (b) Cumulative negative climate hazard refers to the changes in warming, oxygen loss, acidification, and declining food supply (POC flux) relative to the historical variability by 2041–2060. (c) Cumulative negative climate hazard by 2081–2100. The gray polygons in the global map show the extent of the CCZ and MAR, respectively. Projections for the CCZ (left bottom panel in a–c) suggest strong regional variations among exploration contracts (brown), reserve areas (green), and areas of particular environmental interest (APEIs—gray), which are designated no‐mining zones in the CCZ. The right sub‐panels show the exploration contracts (brown) and ridge (gray) within a 150‐mile MAR buffer zone

Over the past half century, the ocean below 200 m has experienced warming (Desbruyères, Purkey, McDonagh, Johnson, & King, 2016), oxygen loss (Breitburg et al., 2018; Oschlies, Brandt, Stramma, & Schmidtko, 2018), and acidification (Chen et al., 2017; Perez et al., 2018). Overall, food supply to the deep seafloor will decrease as a result of climatic changes (Bindoff et al., 2019; FAO, 2019; Sweetman et al., 2017; Figure S2). In many parts of the world ocean, mean changes to environmental conditions already exceed historical variability (annual standard deviation between 1951 and 2000), or will do so by 2100 (FAO, 2019; Figure 1a; Figure S1a; Table 1).

**TABLE 1:** Minimum and maximum values of time of emergence and cumulative negative climate hazard under climate scenarios RCP 8.5 and 2.6 for four climate drivers (temperature, O_2_, pH, and POC flux) in the global ocean, Clarion Clipperton Zone (CCZ), and Mid‐Atlantic Ridge (MAR)

Area	RCP 8.5		RCP 2.6	
	Min	Max	Min	Max
Time of emergence for all four climate drivers				
Global	2023	>2100	2022	>2100
CCZ	2028	>2100	2033	>2100
MAR	2031	>2100	2036	>2100
Cumulative negative climate hazard by 2041–2060				
Global	0.0	67.4	0.0	63.6
CCZ	6.3	38.5	6.0	36.6
MAR	8.9	24.9	8.7	20.7
Cumulative negative climate hazard by 2081–2100				
Global	0.0	148.2	0.7	113.1
CCZ	13.5	105.1	12.7	102.4
MAR	14.8	45.7	11.2	39.1

Given that biological communities adapt and/or acclimate to long‐term stability or variability of environmental conditions (Smit, Burton, Klein, & Wandel, 2000), we refer to the ratio of modeled climate change to historical variability as “Climate Change Hazard.” The historical variability is defined as the standard deviation of annual climate conditions modeled between 1951 and 2000. Areas currently subject to deep bottom fishing (slopes and seamounts 200 to ~2,500 m) and those targeted for deep‐seabed mining are all projected to experience changing environments under both high‐emission (representative concentration pathway [RCP] 8.5; Figure 1; Table 1) and low‐emission (RCP 2.6; Figure S1; Table 1) scenarios; see Supplement S1 (Bindoff et al., 2019; FAO, 2019). In the CCZ, a region of the eastern Pacific Ocean where 18 countries hold 16 mining exploration contracts, and on the northern MAR where several mining exploration contracts exist around hydrothermal vents, multiple climate variables over large regions are projected under the RCP 8.5 scenario to see future variability exceeding historical variability by 2030 (termed the “Time of Emergence”; ToE; Figure 1a; Figure S2; Table 1), a period well within potential 20‐ to 30‐year mining exploitation contracts. Under RCP 2.6, times of emergence for the CCZ and MAR are later, but within this century (Figure S1a; Figure S2). Globally, under both RCP 8.5 and RCP 2.6, the cumulative negative hazard in the ocean represented by accumulated warming, oxygen loss, acidification, and food shortfall (Figure S2) will approach 60 times their summed modeled historical variability by 2041–2060 and more than (RCP 8.5) or nearly double (RCP 2.6) that by 2081–2100 (Figure 1b,c; Figure S1b,c). For the CCZ and MAR, projected cumulative negative hazard under RCP 8.5 and 2.6 will exceed 6–38 times modeled historical variability by 2041–2060 (Figure 1b; Figure S1b; Table 1), and 14–105 times by 2081–2100 (Figure 1c; Figure S1c; Table 1).

Mining, climate change, and fishing impacts will interact. For example, much of the northern MAR (about 84%) overlaps areas managed by the North East Atlantic Fisheries Commission (NEAFC) RFMO (Figure S3b,d). This area may see climate ToE as soon as 2032 under RCP 8.5 and 2036 under RCP 2.6, and experience 15–45 times or 11–32 times the cumulative negative climate change hazard by 2081–2100 under RCP 8.5 and 2.6, respectively (Figure 1; Figure S3). Noting intensive trawling activity in the region, bottom trawlers could potentially fish approximately 28% of these depths (1,000–2,500 m; Dunn, Jablonicky, et al., 2018; Figure S3), suggesting potential cumulative impacts with mining and climate change. The MAR also accounts for 76% of vulnerable marine ecosystems (VMEs) designated by NEAFC; these VMEs, identified as fragile areas protected from bottom fishing, are projected to experience ToE as early as 2031 (2037) and 19–44 times (13–29 times) cumulative negative hazards by 2081–2100 under RCP 8.5 (RCP 2.6) (Figure S3b,d).

## 3 CONSEQUENCES OF CLIMATE IMPACTS ON DEEP‐SEA COMMUNITIES

### 3.1 Climate change impacts on recovery from disturbance

Projected changes in climate drivers will affect deep‐seafloor populations in ways that lead to range shifts (Brito‐Morales et al., 2020), loss of suitable habitat (e.g., Morato et al., 2020), decreases in food availability and biomass (Jones et al., 2014), reduction in growth and reproduction (e.g., Hennige et al., 2015), and ultimately biodiversity decreases (e.g., Sperling, Frieder, & Levin, 2016). Warming will (a) raise metabolic rates and energy requirements, likely increasing food limitation, (b) increase oxygen requirements, threatening recovery in regions with declining oxygen through increased stratification, and/or (c) bring thermal conditions that exceed many species' tolerance ranges (Levin, 2019; Yasuhara & Danovaro, 2016). Contemporary and future species range shifts resulting from deep‐water warming are expected to exceed shallow‐water shifts, even under RCP 2.6 (Brito‐Morales et al., 2020).

Reduced carbonate saturation states (Sulpis et al., 2018) may lower calcification rates, or at least require deep‐sea corals, molluscs and other calcifying fauna to expend more energy to maintain their carbonate structures (Tittensor et al., 2010), and thus exacerbate the impacts of reduced food supply. Declining pH will increase dissolution of non‐living parts of carbonate reefs, the massive mounds of cold‐water coral rubble that accumulate, and provide important deep‐sea habitats (Carreiro‐Silva et al., 2014; Gehlen et al., 2014; Hennige et al., 2015). Declines in particulate organic carbon (POC) flux to the seabed will reduce food supply in oligotrophic, already extremely food‐limited environments, reducing benthic biomass (Jones et al., 2014; Wei et al., 2010; Yool et al., 2017) and potentially lowering reproductive and growth rates and slowing recovery from disturbance.

Climate change will likely further slow recovery in seamount and abyssal systems where great longevity and slow growth already impede recovery from fishing or simulated mining disturbances (Clark et al., 2016; Clark, Bowden, Rowden, & Stewart, 2019; Cuvelier et al., 2018; Hiddink et al., 2019; Jones et al., 2017; Simon‐Lledó et al., 2019). Finally, climate change alterations to food webs and oceanographic conditions are likely to increase the bioaccumulation and biomagnification of metals and other contaminants in marine food webs (Alava, Cisneros‐Montemayor, Sumaila, & Cheung, 2018; Schartup et al., 2019), exacerbating mining‐related releases of metals or other contaminants (e.g., suspended sediments) that may ultimately affect our seafood supply (Drazen et al., 2019).

Nearly all seafloor habitats, including chemosynthetic habitats such as hydrothermal vents, support species with planktonic larvae that disperse via the upper water column; changing water conditions could impact the fitness and survival of these larvae (FAO, 2019). Additionally, warming will alter ocean circulation, changing larval transport and altering the ability of populations to recolonize disturbed areas and recover. The slowing Atlantic Ocean circulation (Srokosz & Bryden, 2015), increasing acidification (Perez et al., 2018) and spreading of corrosive waters to deep‐sea areas of the MAR, will certainly contribute to such effects. Climate variability may negatively influence larvae that disperse higher in the water column, in particular. For example, whereas enhanced subtropical gyre circulation may extend dispersal distances (Collins et al., 2010), reduced equatorial currents (Roemmich et al., 2007), and Atlantic meridional overturning circulation (Srokosz & Bryden, 2015) may limit dispersal ranges. Changes in circulation could alter trajectories of sediment plumes generated by deep‐seabed mining, trawling, or other impacts, affecting adjacent contract areas, closures, EEZs, or protected areas, and altering the effectiveness of networks of marine protected areas (MPAs) (Fox, Henry, Corne, & Roberts, 2016).

Both bottom trawling and seabed mining create a large footprint of physical and chemical disruption on the seabed that includes loss of fauna including structure‐forming foundation species, burial or smothering, release of contaminants, extraneous sound, light, and vibration, altered habitat suitability, and altered or novel species interactions (Clark et al., 2019; Jones et al., 2017; Levin et al., 2016; Pusceddu et al., 2014; Simon‐Lledó et al., 2019). Differentiating impacts directly attributable to extractive activities from those generated by climate change will require essential steps. Baseline data collected prior to extraction should accurately characterize environmental conditions, natural variability, and ecosystem attributes subject to climate change. Furthermore, local and regional baseline observations improve and help validate regional climate model projections (environmental data), habitat suitability models (faunal distribution and environmental data), and larval connectivity models (current data, genetic data), whose outputs can help avoid, mitigate, and reduce impacts. Incorporating climate change into management of deep‐sea use should enhance understanding of impacts and could ultimately reduce industry liability.

### 3.2 Could resource extraction affect carbon cycling?

Several aspects of large‐scale bottom disturbance (e.g., from mining of polymetallic nodules or bottom trawling) could potentially affect deep‐sea biota, though with uncertain feedbacks on global carbon cycling. Bottom trawling and mining resuspend sediments, reducing seabed carbon storage and enhancing water‐column remineralization (Atwood, Witt, Mayorga, Hammill, & Sala, 2020; Legge et al., 2020). However, in shallower environments released nutrients could stimulate primary production and carbon drawdown; the magnitudes and net effects of these changes remain uncertain (Legge et al., 2020). Trawling or mining may disrupt dark (non‐photosynthetic) carbon fixation associated with deep‐sea water, sediments, or nodules (Sweetman et al., 2019), with consequences for local carbon cycling (Orcutt et al., 2020).

The deep sea below 1,000 m holds more than 80% of global marine carbon stocks (Atwood et al., 2020) and recovery of abyssal microbes and habitats could require decades to centuries following disturbance (Stratmann et al., 2018; Vonnahme et al., 2020). Nevertheless, abyssal disturbance‐induced remineralization is unlikely to influence atmospheric CO_2_ concentrations in the near future given the low concentration and refractory nature of carbon in sediments (Orcutt et al., 2020) and millennial time scales of carbon cycling at those depths (Atwood et al., 2020).

Animals play key roles in carbon processing (Snelgrove, Thrush, Wall, & Norkko, 2014); disturbance‐induced declines in animal density and biomass could reduce rates of carbon removal from the biosphere by reducing animal‐mediated mixing and burial of carbon (bioturbation) into the sediments that cover most of the deep‐sea floor. Anticipated increases in water temperature, even at great depths, will enhance rates at which all organisms decompose organic material, increasing remineralization and reducing carbon burial, thereby potentially altering global carbon cycles over long time scales. However, based on existing information, we cannot conclude that feedbacks from DSM itself will significantly impact global carbon cycling.

Particulates (Passow & De La Rocha, 2006) or chemicals (Passow, 2016) released in the water column by mining or trawling can combine with organic material, altering material flux to the deep sea, potentially altering carbon sequestration (Pabortsava et al., 2017). They might enhance oxygen depletion in the water column, potentially enhancing the production of greenhouse gases such as nitrous oxide or reducing active vertical carbon transport by reducing abundances of vertically migrating fauna.

Mining at hydrothermal vents or seamounts disturbs a much smaller area of seabed than mining of abyssal nodules, and seems unlikely to create climate feedbacks, although vent emissions moderate iron supply for surface production (Tagliabue et al., 2017). Nevertheless, localized currents along mid‐ocean ridges (Lahaye et al., 2019) and seamounts may enhance mining plume dispersal and its impacts. Limited information on deep‐sea carbon‐cycling processes constrains certainty regarding the magnitude of mining effects; however, current evidence on rates suggests modest overall effects. Relative to mining, bottom trawling may be more disruptive to carbon sequestration because it occurs at bathyal depths on continental margins, where significant carbon burial occurs (Atwood et al., 2020).

## 4 MANAGEMENT OF HUMAN USE OF DEEP‐SEA RESOURCES SHOULD ADDRESS CLIMATE CHANGE

### 4.1 The case of deep‐seabed mining

We suggest that protection of the marine environment should involve understanding of climate‐change impacts in areas targeted by deep‐seabed mining, and how these changes will interact with mining impacts to affect biodiversity, ecosystem functions, and resulting ecosystem services. The management of deep‐seabed mining, including by the ISA, should consider climate change within regulations for international and national exploration and exploitation.

Current ISA exploration regulations include no requirement for climate change considerations (e.g., ISBA/20/A/9; http://undocs.org/ISBA/20/A/9), although newly revised guidance for environmental impact assessment (ISBA/25/LTC/6/Rev.1; http://undocsorg/ISBA/25/LTC/6/Rev.1) recommends establishing an environmental baseline study against which to compare background variability, climate change and impacts caused by mining activities, and indicates deep waters brought to the surface should not allow the degassing of climate‐active gases. The current ISA Draft Exploitation Regulations (ISBA/25/C/WP.1 March 2019; http://undocsorg/ISBA/25/C/WP.1) include guidance for environmental impact assessment (Annex IV 4.11 Greenhouse gas emissions and climate change), calling for a description of the level of gas and chemical emissions from both natural and anthropogenic activities in the Area, as well as those affecting seafloor and water‐column chemistry. The ISA Strategic Plan (ISBA/24/A/10; http://undocsorg/ISBA/25/A/10) also refers to UN Sustainable Development Goal 13 (*Take urgent action to combat climate change and its impacts*) by suggesting specific research programs designed to assess essential ecological functions of the deep ocean through long‐term, underwater oceanographic observatories in the Area. However, the ISA high‐level action plan and priorities document includes no strategic focus or specific action plan for climate change (ISA, 2019).

The ISA, following calls by the UN General Assembly, is committed to formulating Regional Environmental Management Plans (REMPs) to address potential impacts of mining‐related activities on marine environments. Despite ongoing efforts to develop REMPs for cobalt‐rich crusts in the northwest Pacific Ocean and massive sulfide deposits on the northern MAR, the ISA has approved and promulgated only one environmental management plan to date, in 2012, which addresses the CCZ abyssal polymetallic‐nodule fields (ISBA/17/LTC/7; http://undocsorg/ISBA/17/LTC/7). The CCZ goals include the following: (a) facilitating environmentally responsible exploitation of seabed mineral resources; (b) helping halt biodiversity loss, establishing ecosystem approaches to management, and developing MPAs; (c) maintaining regional biodiversity, ecosystem structure, and ecosystem function; (d) managing the region consistent with integrated ecosystem‐based management; (e) enabling the preservation of representative and unique ecosystems; (f) using (“capitalize on”) available data; (g) environmental monitoring; and (h) promoting cooperative research to better understand environmental conditions in the region to inform future rules, regulations, and procedures. Here, we argue that climate change will affect achievement of each of these goals.

In the CCZ environmental management plan, recommendations from the ISA Legal and Technical Commission on Areas of Particular Environmental Interest (APEIs) include the following: *How to encourage the inclusion of the area of particular environmental interest sites as reference areas in scientific research programmes on climate change and the oceans* (ISBA/17/LTC/7, item 43c; http://undocsorg/ISBA/17/LTC/7). Beyond this, we suggest APEI design should incorporate future climate effects on environmental conditions (e.g., Dunn, Van Dover, et al., 2018), larval transport, and habitat suitability. The development of area‐based management tools within REMPs could reflect climate change through goals such as protecting areas with minimal absolute or relative modeled changes (as climate refugia), avoiding, reducing, and mitigating climate change impacts to facilitate adaptation, and including the full spectrum of future conditions in a protected area network to ensure representative conservation of all ecosystem types. For example, our analyses of the CCZ suggest that current APEIs will not function as better climate refugia in the future, nor add resilience beyond the exploration, reserve, and background areas for most climate variables (Figure 1b,c; Figure S4).

Further development and application of specific metrics that incorporate climate change into spatial planning is needed; these could include absolute or relative change in temperature, oxygen, food availability, and pH (e.g., Dunn, Van Dover, et al., 2018; Table S3), ToE of those variables from background natural variability (e.g., Mora et al., 2013; Table S1), local climate velocity (the distance between a current location and its future climate analog; e.g., Brito‐Morales et al., 2018, 2020), climate hazard (e.g., FAO, 2019; Table 1; Table S2), and species‐specific climate refugia (Keppel et al., 2012; Morato et al., 2020). Ongoing REMP development for the northwest Pacific Ocean and northern MAR offer an excellent opportunity to employ these metrics.

### 4.2 Data and models to predict climate‐change impacts on ecosystems for management

Despite the addition of significant data and knowledge on the distribution and biology of select deep‐sea species by recent scientific exploration, these ecosystems remain poorly known, with few sustained, long‐term measurements of environmental conditions (Bindoff et al., 2019). These gaps point to the critical importance of mathematical modeling tools in forecasting climate‐change impacts on deep‐sea ecosystems and incorporating climate change into deep‐sea management plans. We illustrate climate projection metrics based on Earth‐System Models above (Figure 1; Figures S1 and S2). Below, we highlight several ecological modeling approaches with potential to inform key management issues.

#### 4.2.1 Modeling to inform impacts on larval transport

The maintenance of populations critically depends on connectivity, which is enabled at the front end by larval dispersal, and at the back end by suitable habitat. Protecting ecological connectivity can reduce long‐term impacts of human activities on marine communities, as well as risks from climate change (Balbar & Metaxas, 2019). Thus, assessment of disturbance effects on populations, regional planning, and effective design of protected, reference, and impact areas depends on underlying knowledge of connectivity.

**FIGURE 2:**
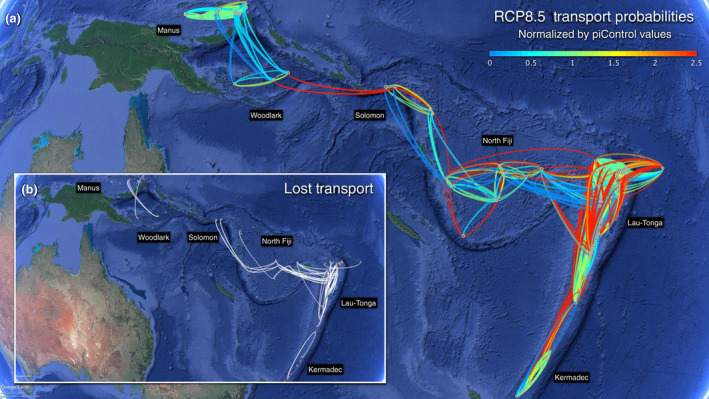
Global warming causes losses and increases of potential larval transport among hydrothermal vents in the Southwestern Pacific Ocean. (a) Transport frequencies (how often source and destination vent fields can be connected by ocean circulation) are computed under the RCP 8.5 scenario from years 2090 to 2099, normalized by the preindustrial control case. Enhanced transport (>100% increases in transport frequencies) is indicated with red lines, while reduced transport (<50% reductions) is indicated with blue lines. (b) White lines indicate lost transport (potential larval transport present in the preindustrial control case that vanishes under the RCP 8.5 scenario). Transport frequencies from all active vent fields in the western Pacific Ocean were assessed using a regional ocean modeling system nested within the CMIP5 coupled global climate models. We set dispersal depth to 1,000 m below the sea surface. Transport time is set to 180 days, a period long enough to cover larval development times of marine species under the mean water temperature of 5°C at 1,000 m (O'Connor et al., 2007)

Given assumptions that ocean circulation primarily controls deep‐sea larval dispersal (Hilário et al., 2015), climate‐induced changes in circulation could affect the transport of larvae. High‐resolution ocean circulation models can help forecast impacts of climate change on ocean circulation processes under the IPCC future climate change scenarios, capturing advection from basin‐scale ocean currents to mesoscale eddy transport. Ocean circulation models, combined with particle tracking models that simulate larval transport, allow assessment of climate‐change effects on larval dispersal at sufficiently fine spatiotemporal resolution (~km and ~hours) to capture potential larval connections among deep‐sea habitats (Mitarai, Watanabe, Nakajima, Shchepetkin, & McWilliams, 2016). For example, ocean circulation models predict increasingly frequent larval connections between hydrothermal vent fields in the Western Pacific Lau Basin (Southwest vent complex) with global warming under RCP 8.5 (Figure 2a) and RCP 2.6 (Figure S5a), although some connections may disappear (Figure 2b; Figure S5b). Deep penetration of the enhanced South Equatorial Current at mid latitudes could increase some larval connections in this region, whereas ongoing slowing of Atlantic Ocean circulation (Srokosz & Bryden, 2015) may decrease structural connections. While the number of larval connections increased among Western Pacific vents in this example, models generally project an overall decrease in dispersal distance and thus larval connectivity under climate change (Gerber, Mancha‐Cisneros, O'Connor, & Selig, 2014).

Larval dispersal models nested with biological trait information and ocean circulation models can inform on potential climate‐change impacts on larval supply and recruitment, and identify source and sink populations under future conditions. Future larval exchange patterns can inform MPA design to capture areas that help maintain population connectivity. Additionally, dispersal distances (Gerber et al., 2014) can inform MPA design because widely accepted guidelines suggest size and spacing should scale to mean dispersal distance of target species; effective MPAs may require larger sizes and closer spacing to compensate for habitat loss under future reduced structural connectivity (Gerber et al., 2014). However, comprehension of climate‐change impacts on population connectivity will critically depend on further studies of ocean circulation changes, increased knowledge of life‐history traits of deep‐sea species (spawning seasons, ontogenetic vertical migration behaviors, planktonic larval durations, etc.; Hilário et al., 2015) as well as on climate‐ or physical disruption‐induced changes in the suitability of the habitat where larvae settle (e.g., Levin et al., 2016; Morato et al., 2020).

#### 4.2.2 Modeling to inform impacts on species distribution

Spatial redistribution of biodiversity represents a fundamental ecological response to climate change (Brito‐Morales et al., 2018, 2020; Burrows et al., 2014; FAO, 2019). Understanding the processes contributing to species range shifts in response to climate change will critically inform the development of conservation measures, improving the likelihood of delivering the conservation objectives envisioned in their design. Species distributions also underlie changing baselines, environmental impact assessment, and use of indicator taxa for monitoring; effective management requires knowing whether habitats will remain suitable for species.

In conjunction with climate, ocean circulation, and larval dispersal modeling, environmental niche modeling (also known as species distribution modeling, habitat suitability modeling, or climate envelope modeling), assuming species' environmental niches are constant over time, can forecast changes in species' distributions under future climate scenarios (Hattab et al., 2014; Hijmans & Graham, 2006). Previous studies have applied these modeling tools to many marine species and habitats including the deep sea (FAO, 2019; Morato et al., 2020), and generated climate‐relevant metrics to inform area‐based management. Specifically, habitat suitability models can help identify where the predicted present habitat overlaps with projected future suitable habitat and inform MPA placement (Johnson & Kenchington, 2019) on such species‐specific resilient and refugia areas (Keppel et al., 2012).

**FIGURE 3:**
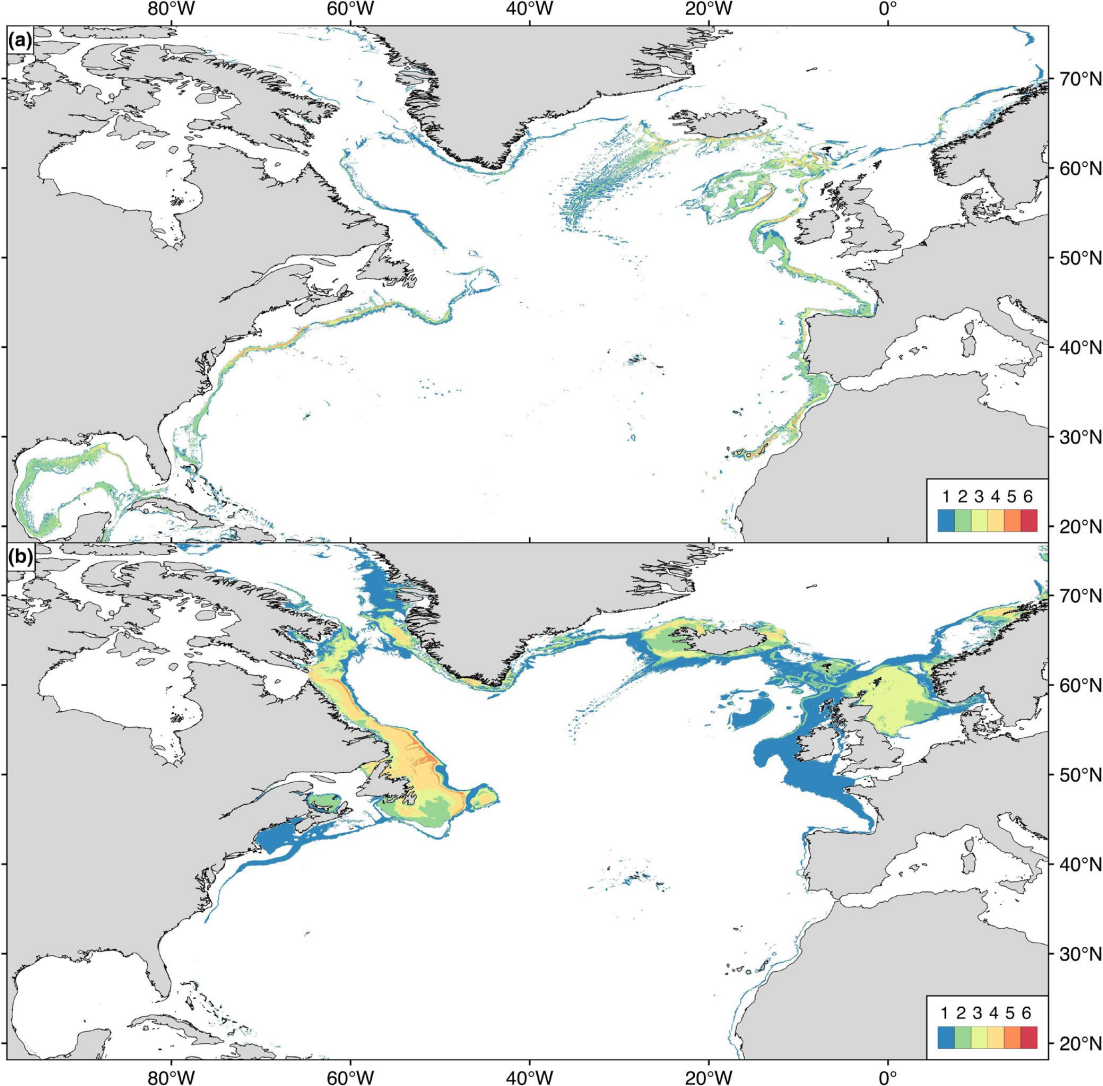
Forecasted cumulative climate refugia areas (sensu Keppel & Wardell‐Johnson, 2012) under future (2081–2100) environmental conditions under RCP 8.5 for (a) six cold‐water corals: 1. *Lophelia pertusa (recently reclassified as Desmophyllum pertusum)*, *2. Madrepora oculata*, 3. *Desmophyllum dianthus*, *4. Acanthogorgia armata*, 5. *Acanella arbuscula*, *6. Paragorgia arborea* and (b) six deep‐water commercially important fishes: 1. *Helicolenus dactylopterus*, 2. *Sebastes mentella*, 3. *Gadus morhua*, 4. *Hippoglossoides platessoides*, 5. *Reinhardtius hippoglossoides*, 6. *Coryphaenoides rupestris)* in the North Atlantic Ocean. Areas were identified from binary maps built with an ensemble modeling approach and the maximum sensitivity and specificity thresholds (MSS). The model outputs used to build Figure3 were published in Morato et al. (2020) 'Areas were identified from binary maps built with an ensemble modeling approach and the maximum sensitivity and specificity threshold (MSS)'

A recent North‐Atlantic study used ensemble environmental niche modeling, best available species occurrence data, and environmental parameters to model habitat suitability for key cold‐water coral and commercially important deep‐sea fish species under present‐day (1951–2000) and future (2081–2100; RCP8.5) environmental conditions (Morato et al., 2020). The model forecasted marked decreases in suitable habitat for many cold‐water coral species and a shift towards higher latitudes for many deep‐water commercially important fishes. The authors also inferred suitable North Atlantic refugia (sensu Keppel & Wardell‐Johnson, 2012) both under present‐day and projected (RCP 8.5) future conditions. By combining such data for multiple species of scleractinian corals and octocorals (Figure 3a) and fishes (Figure 3b), locations are identified that provide assemblage‐level climate refugia. Larval dispersal models can then evaluate the likelihood of species colonizing these areas following seafloor disturbance (e.g., Figure S2; Figure S5; Ross, Wort, & Howell, 2019); a complementarity approach spatial planners and environmental managers can exploit.

## 5 ACHIEVING HOLISTIC MANAGEMENT

### 5.1 Data generation via integration of observing systems

The climate, larval dispersal, and habitat suitability modeling approaches described above require data both for construction and groundtruthing. The large size and remoteness of the deep ocean are partly responsible for the sparsity of environmental, climate, and biological data. Stronger collaboration among scientists, states, regulators, and the private sector can help meet data needs (Levin et al., 2019). The MOU between the ISA and UNESCO IOC, which oversees the Global Ocean Observing System (GOOS) and the largest repository of biological observations of the ocean, the Ocean Biodiversity Information System (OBIS), offers one avenue for collaboration. The Deep Ocean Observing Strategy (DOOS) is currently addressing data deficiencies for societal needs, in part by developing GOOS/DOOS essential ocean variables (EOVs) and demonstration projects (Levin et al., 2019). Coordinated expansion of international observing programs in critical regions, such as the CCZ, can inform on climate change and disturbance impacts in the deep ocean.

Harnessing current computational tools can elicit and accelerate the processing of climate‐relevant data delivered by deep‐ocean platforms, sensors, and high‐throughput technologies such as multi‐omics, imaging, and acoustics. Data‐release strategies would account for the granularity of natural and scientific processes, which occur over multiple temporal and spatial scales. Infrastructure enabling data availability, harmonization, and use should enforce and administer open access to both existing and novel data relevant to spatial planning, decisions about contracts, and impact assessments. The goal of the ISA database, DeepData (www.isa.org.jm/central‐data‐repository) is long‐term mechanisms of curation, standardization and validation. This database should standardize, expand, refine, and diversify its contents to facilitate incorporation of climate‐relevant data. Intense confidentiality restrictions have long constrained biodiversity data from fisheries observer programs despite public funding. However, recent examples illustrate how civil society and policymakers can aggregate biodiversity knowledge (model outputs) without exposing the underlying data (Donald et al., 2019; Dunn et al., 2019). For example, both the Marine Important Bird Area e‐atlas (https://maps.birdlife.org/marineIBAs) and the Migratory Connectivity in the Ocean System (https://mico.eco/system) provide actionable knowledge to managers, industry, and policymakers through geospatial summaries of species observation data (i.e., polygons describing area use). The incredible wealth of information on deep‐sea biodiversity held in fisheries' observer databases could help fill gaps in habitat suitability models that can inform area‐based management and biodiversity conservation in the deep ocean (Dunn, Jablonicky, et al., 2018).

### 5.2 Climate requires cross‐sectoral considerations

Climate change cross‐cuts ocean governance and therefore offers a unifying focus for integrating different multilateral agreements. Governance solutions require comprehensive research to understand climate‐change interactions with direct cumulative effects of human activities in the deep sea, deep‐ocean biodiversity, access to data and information, application of the precautionary approach in decision‐making, assessment of cumulative impacts, strategic environmental assessments (SEAs), better understanding of the role of area‐based management tools (including MPAs) in building resilience to climate change, and cooperation and coordination among institutions. Concurrently, we must ensure the public appreciates implications for trade‐offs and benefits derived from deep‐ocean ecosystem services (e.g., Le, Levin, & Carson, 2017).

Opportunities for incorporating deep‐ocean climate science into policy exist at both regional and global scales. Regional entities with a mandate for sustainable development of deep‐ocean areas (such as selected Regional Seas Conventions and Regional Fisheries Management Organizations) already recognize and address aspects of deep‐ocean climate changes (FAO, 2019) including implications for protected areas and movement of fish stocks (e.g., Cheung et al., 2018). Coordination among governing bodies and their jurisdictions will help ensure protection of the marine environment (Wright et al., 2019). There are multiple international fora currently engaged with processes addressing or taking note of deep‐ocean climate change (Table 2).

**TABLE 2:** International reports or fora addressing or considering climate change relevant to the deep ocean

UNFCCC: The Intergovernmental Panel on Climate Change (IPCC) Special Report on the Ocean and Cryosphere in a Changing Climate inextricably links climate change and the deep ocean (Bindoff et al., 2019) Sectoral marine resource interests Food and Agriculture Organization (fisheries) International Maritime Organization (shipping) International Seabed Authority (mining) International Cable Protection Committee (sub‐sea cables) Biodiversity protection interests Convention on Biological Diversity (CBD) Post‐2020 Global Biodiversity Framework Negotiations for a new international legally binding instrument on conservation and sustainable use of marine biological diversity of areas beyond national jurisdiction (BBNJ) Intergovernmental Science‐Policy Platform on Biodiversity and Ecosystem Services (IPBES) The Convention on the Conservation of Antarctic Marine Living Resources (CCAMLR) Scientific research plans UN Decade of Ocean Science for Sustainable Development Global Ocean Observing System Deep Ocean Observing Strategy

Climate considerations are materializing just as unprecedented rates of biodiversity loss occur, with “knock‐on” consequences for ecosystem goods and services (Pecl et al., 2017). In addition to provisioning services (i.e., fish, energy, minerals), climate change potentially threatens other deep‐sea ecosystem services including genetic resources (Harden‐Davies, 2017), bioremediation (Teske, 2019), and other supporting services necessary for continual benefit from these habitats. Recognizing this threat, the significance of active biological communities for carbon sequestration and the need to better understand how different biological components contribute to ecosystem services have been highlighted recently by Rogers (2020) and Chami, Cosimano, Fullenkamp, and Oztosun (2019).

### 5.3 A mechanism to ensure appropriate climate‐change considerations

Strategic environmental assessment, hitherto not applied within areas beyond national jurisdiction (ABNJ), offers an appropriate tool at a regional or ecosystem‐level scale, to ensure holistic and proactive cross‐sectoral consultation (Jones et al., 2019). Regional‐scale environmental assessment offers a valuable tool for managing broad‐scale and cumulative impacts, such as climate change. Applying SEA to regional planning, such as the ISA REMPs, would allow early scoping of climate opportunities and risks, as well as transparent iteration of alternatives based upon appropriate climate predictions and comparisons (Durden et al., 2017). Negotiations for a new treaty under UNCLOS for the conservation and sustainable use of marine biodiversity in ABNJ (UNGA resolution 72/249) could include provisions for collecting, sharing, and updating scientific information at an appropriate scale, including for deep‐ocean climate parameters. The negotiations (third substantive session, draft text note by the President, A/CONF.232/2019/6, Art. 28) are considering SEA, but as of writing, we feel it is premature to predict if and how they will include this.

### 5.4 Research and capacity gaps and science priorities

Improved incorporation of climate change into the management of deep‐ocean activities requires research, and could be incorporated into the forthcoming UN Decade of Ocean Science for Sustainable Development (2021–2030). Priorities include the following: (a) expanded discovery and characterization of new species; (b) acquiring life history and other critical data for key or indicator species to inform connectivity, biological traits, and assess vulnerability to climate change; (c) improved understanding of animal thresholds and sensitivity to change, potentially through studies across natural gradients or via laboratory experiments; (d) information about ecosystem functions of deep‐sea taxa, how climate change affects them, and how these changes alter ecosystem services; and (e) quantitative carbon cycle data: carbon burial, POC flux data, rates of chemoautotrophic carbon‐fixation, biological mineralization, water‐column fluxes, release of carbon (e.g., Dissolved Organic Carbon) by viral lysis, etc.

Groundtruthing of climate projections, larval dispersal, and habitat suitability models require high‐resolution, in situ data (environmental, bathymetric, and faunal distributions). A modeling intercomparison (or cross talk) project for deep‐sea biodiversity could combine climate modeling, physical modeling, impact modeling of connectivity, and faunal distributions with additional biological expertise, for example, in population genetics and biological traits. Maintaining ecosystem integrity will also critically require understanding microbial responses to climate change in tandem with physical disturbance (Orcutt et al., 2020). Multi‐omics offers potential to assess functional diversity (Ansorge et al., 2019) and microbiological control on greenhouse gases (Birrer et al., 2019). Genomics has begun elucidating microbial processes important in nodule‐rich DSM areas (e.g., metal resistance, Gillard, Chatzievangelou, Thomsen, & Ullrich, 2019), whose interaction with climate‐induced change (e.g., acidification, Nikinmaa, 2013) will likely disrupt ecological processes.

Limited global capacity exists to execute the strategies recommended here to incorporate climate consciousness into deep‐sea use and management. Engagement of all countries requires additional infrastructure, technical support, and knowledge transfer. Establishment of international training centers for states lacking the capacity can help standardize methodologies and broaden use of the modeling approaches illustrated above.

## 6 CONCLUSION

Given how climate could alter ecosystems targeted for extractive activities, act as a cumulative stressor, and potentially confound impacts associated with resource extraction, we propose a series of basic principles and practices to adopt in managing deep‐sea industries and activities.

1. Recognize natural variability and ongoing climate change by incorporating climate projections and system connections into standards and guidelines, regulations, environmental objectives, assessment, monitoring, and adaptive management (e.g., in the ISA Mining Code).
2. Design (and implement) suites of area‐based management tools, including MPAs, and develop monitoring programs to progress understanding of direct disturbance impacts (e.g., from resource extraction or geoengineering) as distinct from climate impacts, and to protect the marine environment, using appropriate indicators, measured on climate‐relevant spatial and temporal scales.
3. Incorporate how climate change interacts with direct seabed disturbance to alter ecosystem services into a full accounting of costs of deep‐sea extractive activities.
4. Build capacity to incorporate climate consciousness into the environmental management of deep‐sea use. Develop international training centers to standardize methodologies and strengthen and integrate their capacity to use diverse models (e.g., for climate projections, larval dispersal, and habitat suitability).
5. Require open access to climate‐relevant data (environmental and biological), and enable groundtruthing of climate models in regions targeted for mining.

## Supporting information

Fig S1‐S6

Table S1‐S3

Supplementary Material

## Data Availability

All climate analyses (Figure 1; Figures S1–S4) were conducted using publicly available software and packages from the R Project for Statistical Computing (https://www.r‐project.org/), namely the R packages devtools, ncdf4, raster, abind, gstat, pracma, rgdal, mapdata, maps, maptools, sp, and dplyr. The ensemble average (across three models) of climate data, climate changes, exposure to climate hazards, and negative cumulative impact for the past (1951–2000) and future projections (2041–2060, 2081–2100), as well as the ToE of climate change are available through the GitHub software development platform (https://github.com/). The model outputs are also available for download as R data packages SCC26 (for RCP2.6) and SCC85 (for RCP8.5) from the GitHub (https://github.com/chihlinwei/SCC26 and https://github.com/chihlinwei/SCC86) and can be installed in R using the following commands: install_github ("chihlinwei/SCC26") or install_github ("chihlinwei/SCC85"). For Figure 2, the SRTM30_PLUS bathymetry data used for the ocean circulation model are publicly available at https://topex.ucsd.edu/WWW_html/srtm30_plus.html. The MRI‐CGCM3 model products can be downloaded from a Coupled Model Intercomparison Project 5 (CMIP5) website, for example, https://esgf‐node.llnl.gov/projects/cmip5/. The InterRidge Vent database is publicly available at http://vents‐data.interridge.org. The model outputs used to build Figure 3 were published in Morato et al. (2020) and are available for download from PANGAEA (https://doi.org/10.1594/PANGA EA.910319).
