# Supplementary material for: Climate change considerations are fundamental to management of deep‐sea resource extraction
Source: Glob Chang Biol. 2020 Jul 6;26(9):4664–78. doi: 10.1111/gcb.15223 (PMC7496832; doi:10.1111/gcb.15223)
Supplement: Supplementary file 1 — Fig S1‐S6 [file GCB-26-4664-s001.docx]

**Figure S1.** Climate projections for the RCP 2.6 scenario for the global seafloor and two regions targeted for deep-seabed mining, the Clarion Clipperton Zone (CCZ) (left panels) and the northern Mid Atlantic Ridge (MAR right panels). (a) Time of Emergence: the year when future variability exceeds historical variability for all climate changes in temperature, oxygen, pH and food supply exceed historical variability (i.e., annual standard deviation between 1951 and 2000). (b) Cumulative negative climate hazard by 2041-2060. This refers to the changes in warming, oxygen loss, acidification, and declining food supply (POC flux) relative to the historical variability. (c) Cumulative negative climate hazard by 2081-2100. The gray polygons in the global map show the extent of the CCZ and MAR, respectively. Projections for the CCZ (left bottom panel in a,b,c) suggest strong regional variations among exploration contracts (brown), reserve areas (green) and areas of particular environmental interest (APEIs - grey), which are designated no-mining zones in the CCZ. The right sub-panels show the exploration contracts (brown) and ridge (gray) within a 150-mile MAR buffer zone.

#

# **Figure S2.** Climate change hazard by 2081-2100 under RCP 8.5 (a-d) and RCP 2.6 (e-h) for individual climate drivers in the CCZ (top) and MAR (bottom). Climate change hazard refers to the ratio between climate changes (difference between 2081-2100 and 1951-2000) and historical variability (standard deviation in 1951-2000).

# Figure S3. Climate projections under RCP 8.5 (upper) and RCP 2.6 (lower) for the Clarion Clipperton Zone (CCZ; (a) and (c) and mid-Atlantic Ridge (MAR; (b) and (d). The brown polygons show intersections between NPFC (North Pacific Fisheries Commission) and CCZ and between NEAFC (Northeast Atlantic Fisheries Commission) and MAR respectively. The green polygons show VME (vulnerable marine ecosystem) designated by NEAFC.


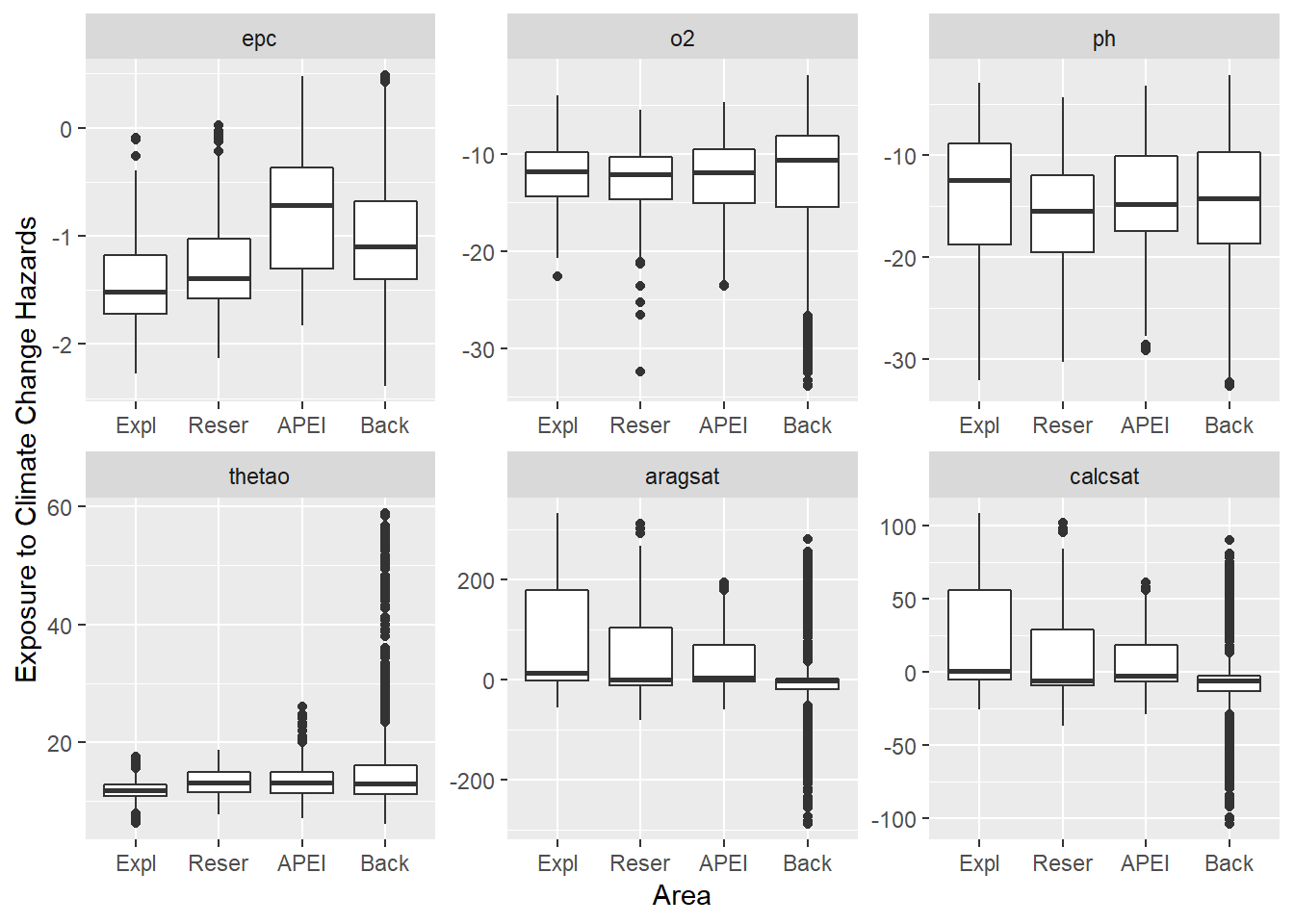


Figure S4. Exposure to climate change hazard for 6 climate drivers in the Clarion in exploration areas (Expl), reserve areas (Reser), Areas of Particular Environmental II) and all other areas (Back) in the Clarion Clipperton Zone. Figures show mean, 25%/75% quantiles, 95% CI and outliers for POC flux (epc), oxygen, pH, temperature (thetao), aragonite saturation state (aragsat) and calcite saturation state (calcsat).

Figure S5.  Potential larval transport between hydrothermal vent fields in the Western Pacific (Southwest vent complex) with global warming. (A) Transport frequencies (how often source and destination vent fields can be connected by ocean circulation) are computed under the RCP 2.6 scenario from years 2090-2099, normalized by the preindustrial control case. (B) White lines indicate lost connections transport (connections potential larval transport present in the preindustrial control case that vanishes under the RCP 2.6 scenario). See the Fig. 2 legend for detailed descriptions.


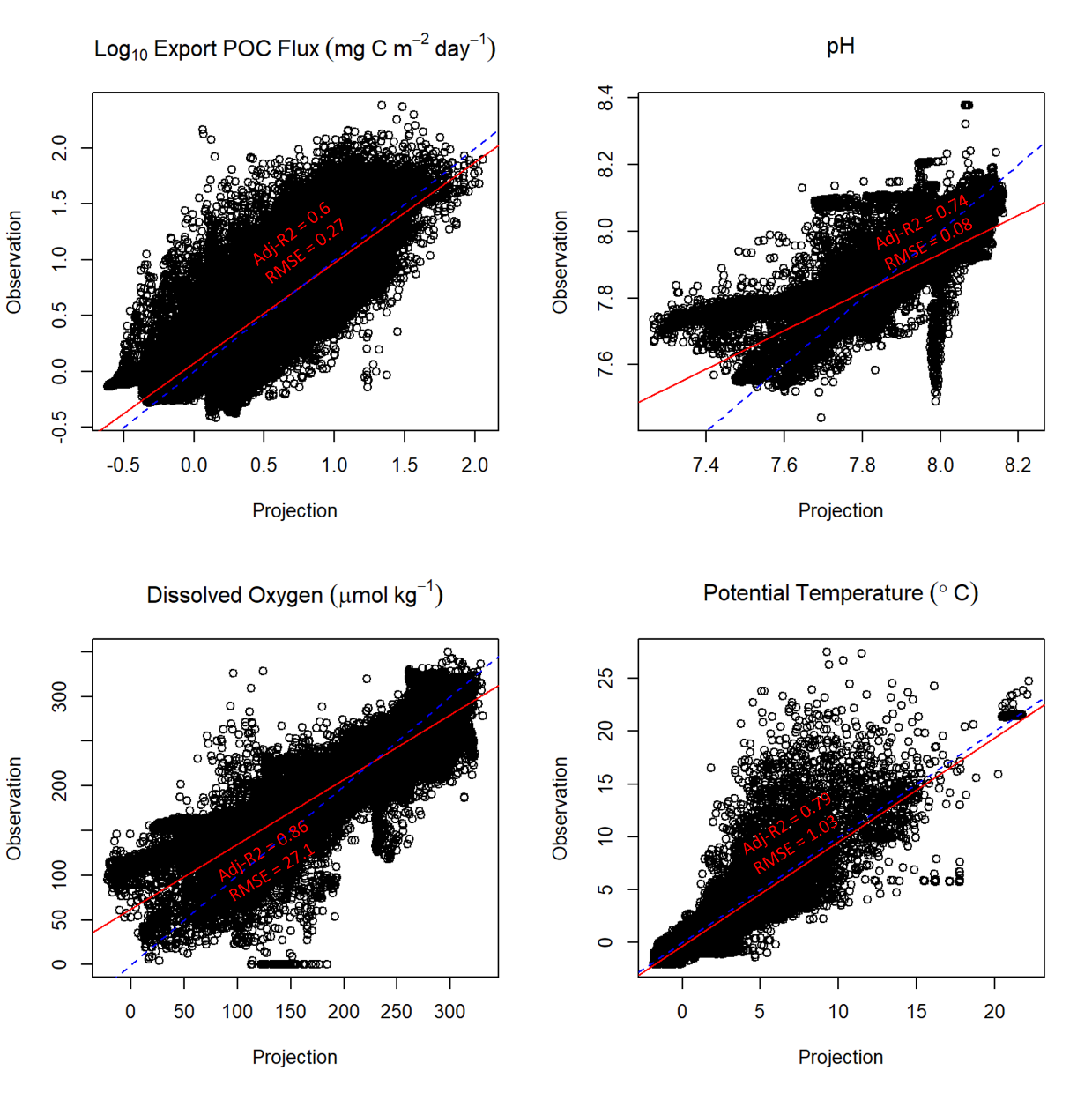


Figure S6. Evaluation of the CIMP5 historical projections (1951-2000) at the seabed against field observations. The correlation between the projected and observed climatological mean were evaluated with the root-mean-square error (RMSE) and the Adjusted R-squared (red lines). Blue dashed lines show 1:1 relationship.
