# Supplementary material for: Climate change considerations are fundamental to management of deep‐sea resource extraction
Source: Glob Chang Biol. 2020 Jul 6;26(9):4664–78. doi: 10.1111/gcb.15223 (PMC7496832; doi:10.1111/gcb.15223)
Supplement: Supplementary file 3 — Supplementary Material [file GCB-26-4664-s003.docx]

# **Supplement 1. Methods for climate projections.**

**Model projections:** We used three 3-D, fully coupled earth system models including the Geophysical Fluid Dynamics Laboratory's ESM 2G model (GFDL‐ESM‐2G; Dunne et al., 2012), the Institut Pierre Simon Laplace's CM6‐MR model (IPSL‐CM5A‐MR; Dufresne et al., 2013) and Max Planck Institute's ESM‐MR model (MPI‐ESM‐MR; Giorgetta et al., 2013). All models are part of Coupled Models Intercomparison Project Phase 5 (CMIP5) and were downloaded from Earth System Grid Federation (ESGF) Peer-to-Peer (P2P) enterprise system (<https://esgf-node.llnl.gov/>). Within each model output, we extracted the downward flux of particle organic carbon at 100-m depth, seawater potential temperature, dissolved oxygen concentration, and pH value for the historical time period from 1951 to 2000 and representative concentration pathways RCP2.6 and RCP8.5 until 2100. The monthly export production at 100 m and temperature were averaged by the year and the bottom most grids from each layer of temperature, dissolved oxygen and pH were retained to construct seafloor environmental conditions. The export production at 100 m was converted to export POC flux at the seafloor (*epc*) using the Martin curve (Martin, Knauer, Karl, & Broenkow, 1987) following the equation: *POC flux* = *export production**(depth/export depth)^0.858^. The export depth was set to 100 m and water depth using the ETOPO1 Global Relief Model (Amante and Eakins, 2008). We then re-projected these bottom grids to 0.5 by 0.5-degree resolution based on bilinear interpolation. The ensemble average (across the three models) of the CMIP5 historical projections (1951-2000) were evaluated against the observed export POC flux based on Lutz, Caldeira, Dunbar, & Behrenfeld (2007) algorithm and monthly ocean productivity data between 1997 and 2007 (<http://sites.science.oregonstate.edu/ocean.productivity/>), decadal mean bottom temperature and oxygen between 1954 and 2017 from World Ocean Atlas 2018 (Locarnini et al., 2018; Garcia et al., 2018), and bottom pH values from Global Ocean Data Analysis Project (GLODAP) version 2 (Lauvset et al., 2016). The in-situ bottom temperature was converted to potential temperature, considering the salinity and pressure at the same location. Model evaluation on the global seafloor data (>200-m depth) show that the CMIP5 historical projections track the observations reasonably well (adjust R^2^ = 0.6-0.86, Figure S6). When only the CCZ seafloor is considered, the correlations between the projected and observed POC flux, dissolved oxygen and temperature are comparable to the global dataset (adjust R^2^ = 0.69-0.86), but the correlations for pH is low (adjust R^2^ < 0.01). The same analysis on the MAR seafloor, however, showed good agreement between the projected and observed values for the four variables considered (adjust R^2^ = 0.56-0.84).

**Data analysis:** We calculated the average and standard deviation of export POC flux, dissolved oxygen, temperature, and pH by year for 1951 to 2000, 2041 to 2060, and 2081 to 2100. We quantified the climate change by subtracting the historical average (1951-2000) from the future average (2041-2060 or 2081-2100). Exposure to climate change hazard is defined as the ratio between climate change and historical standard deviation, measuring the amplitude of climate change in the unit of past variability. By converting the climate changes of export POC flux, dissolved oxygen, temperature, and pH to the units of their own past variabilities, we summed these standardized climate change ratios (or exposure to hazard) to a cumulative impact of climate change, assuming the effects by different variables are additive. We only summed the exposure to hazards associated with decreasing export POC flux, deoxygenation, warming and acidification, understanding these changes causing cumulative negative impacts to deep-sea benthic communities (Mora et al., 2013, Sweetman et al., 2017). Based on the same concept, we calculated the yearly standard deviation for the future period cumulatively from 2006. When the future cumulative standard deviation exceeds the historical standard deviation (1951-2000), we define it as the time of emergence (*ToE*) of the climate changes. We also calculated the overall *ToE* when future cumulative variability of all variables (i.e., export POC flux, dissolved oxygen, temperature, and pH) exceed their past variability.

References:

Amante, C., & Eakins, B. W. (2008). ETOPO1 1 Arc-Minute Global Relief Model: Procedures, Data Sources and Analysis. NOAA Technical Memorandum NESDIS NGDC-24. National Geophysical Data Center, NOAA.

Dufresne, J.-L., Foujols, M.-A., Denvil, S., Caubel, A., Marti, O., Aumont, O., … Vuichard, N. (2013). Climate change projections using the IPSL-CM5 Earth System Model: from CMIP3 to CMIP5. *Climate Dynamics, 40*(9), 2123–2165. https://doi.org/10.1007/s00382-012-1636-1

Dunne, J. P., John, J. G., Adcroft, A. J., Griffies, S. M., Hallberg, R. W., Shevliakova, E., … Zadeh, N. (2012). GFDL’s ESM2 Global Coupled Climate–Carbon Earth System Models. Part I: Physical Formulation and Baseline Simulation Characteristics. *Journal of Climate, 25*(19), 6646–6665. https://doi.org/10.1175/JCLI-D-11-00560.1

Garcia, H. E., Weathers, K., Paver, C. R., Smolyar, I., Boyer, T. P., Locarnini, R. A., … Reagan, J. R. (2018). *World Ocean Atlas 2018, Volume 3: Dissolved Oxygen, Apparent Oxygen Utilization, and Oxygen Saturation*. A. Mishonov Technical Ed.; NOAA Atlas NESDIS 83, 38 pp.

Giorgetta, M. A., Jungclaus, J., Reick, C. H., Legutke, S., Bader, J., Böttinger, M., … Stevens, B. (2013). Climate and carbon cycle changes from 1850 to 2100 in MPI-ESM simulations for the Coupled Model Intercomparison Project phase 5. *Journal of Advances in Modeling Earth Systems, 5*(3), 572–597. https://doi.org/10.1002/jame.20038

Lauvset, S. K, Key, R. M., Olsen, A., van Heuven, S., Velo, A., Lin, X., … Watelet. S. (2016). A new global interior ocean mapped climatology: the 1°x1° GLODAP version 2. *Earth System Science Data, 8*, 325–340. doi:10.5194/essd-8-325-2016

Locarnini, R. A., Mishonov, A. V. , Baranova, O. K., Boyer, T. P., Zweng, M. M., Garcia, H. E., … Smolyar, I. (2018). World Ocean Atlas 2018, Volume 1: Temperature. A. Mishonov Technical Ed.; NOAA Atlas NESDIS 81, 52 pp.

Lutz, M. J., Caldeira, K., Dunbar, R. B., & Behrenfeld, M. J. (2007). Seasonal rhythms of net primary production andparticulate organic carbonflux to depth describe theefficiency of biological pump in the global ocean. *Journal of Geophysical Research,112*, C10011. doi:10.1029/2006JC002706

Martin, J. H., Knauer, G. A., Karl, D. M., & Broenkow, W. W. (1987). VERTEX: carbon cycling in the northeast Pacific. Deep Sea Research Part A. *Oceanographic Research Papers, 34*(2), 267–285. <https://doi.org/10.1016/0198-0149(87)90086-0>

Mora, C., Wei, C.-L., Rollo, A., Amaro, T., Baco, A. R., Billett, D., … Yasuhara, M. (2013). Biotic and human vulnerability to projected changes in ocean biogeochemistry over the 21st century. PLoS Biology, 11(10), e1001682–e1001682. PubMed. https://doi.org/10.1371/journal.pbio.1001682

Sweetman, A. K., Thurber, A. R., Smith, C. R., Levin, L. A., Mora, C., Wei, C.-L., … Roberts, J. M. (2017). Major impacts of climate change on deep-sea benthic ecosystems. Elementa Science of the Anthropocene, 5(0), 4. https://doi.org/10.1525/elementa.203

**Larval transport Modeling:** The model domain covers all active western Pacific vent fields between 32°N and 36°S registered in the InterRidge vents database (Beaulieu, Baker, German, & Maffei, 2013) discretized with a 10-km horizontal mesh and 40 terrain-following vertical layers. The bottom topography is obtained by interpolating the 30 arc-second global bathymetry grid (SRTM30_PLUS) (Becker et al., 2009). The 3D hydrodynamic Regional Ocean Modeling System (Shchepetkin & McWilliams, 2005; Shchepetkin 2015) is used to integrate rotating primitive equations with a realistic equation of state, nested within the MRI-CGCM3 model (Yukimoto et al., 2012) under the pre-industrial control case and RCP8.5 and RCP 2.6 scenarios. The flow is forced at the sea surface by daily momentum, heat, and freshwater fluxes from the MRI-CGCM3 model. Initial and lateral boundary conditions are determined from the monthly time series of MRI-CGCM3. More than 36,000 model floats are introduced from each of the western Pacific vent fields (10 floats per day for 10 years). Float trajectories are obtained by integrating float velocities with 900-s time stepping. Dispersal probabilities from each vent field were assessed similarly to Mitarai, Watanabe, Nakajima, Shchepetkin, & McWilliams (2016).

Beaulieu, S. E., Baker, E.T., German, C. R., & Maffei, A. (2013). An authoritative global database for active submarine hydrothermal vent fields. *Geochemistry, Geophysics, Geosystems 14*, 4892–4905. https://doi.org/10.1002/2013GC004998

Becker, J. J., Sandwell, D. T., Smith, W. H. F., Braud, J., Binder, B., Depner, J., … P. Weatherall. (2009). Global Bathymetry and Elevation Data at 30 Arc Seconds Resolution: SRTM30_PLUS. *Marine Geodesy, 32*, 355–371. https://doi.org/10.1080/01490410903297766

Mitarai, S., Watanabe, H., Nakajima, Y., Shchepetkin, A. F., & McWilliams, J. C. (2016). Quantifying dispersal from hydrothermal vent fields in the western Pacific Ocean. *PNAS U.S.A., 113*(11), 2976-2981. doi:10.1073/pnas.1518395113.

Shchepetkin, A. F. & McWilliams, J. C. (2005). The regional oceanic modeling system (ROMS): a split-explicit, free-surface, topography-following-coordinate oceanic model. *Ocean Modelling, 9*, 347–404. https://doi.org/10.1016/j.ocemod.2004.08.002

Shchepetkin, A. F. (2015). An adaptive, Courant-number-dependent implicit scheme for vertical advection in oceanic modeling. *Ocean Modelling, 91*, 38–69. <https://doi.org/10.1016/j.ocemod.2015.03.006>

Yukimoto, S., Adachi, Y., Hosaka, M., Sakami, T., Yoshimura, H., Hirabara, M., Tanaka, T. Y., Shindo, E., Tsujino, H., Deushi, M., Mizuta, R., Yabu, S., Obata, A., Nakano, H., Koshiro, T., Ose, T., Kitoh, A. (2012). A New Global Climate Model of the Meteorological Research Institute: MRI-CGCM3 —Model Description and Basic Performance—. 90A, 23–64.
