# Supplementary material for: Climate change considerations are fundamental to management of deep‐sea resource extraction
Source: Glob Chang Biol. 2020 Jul 6;26(9):4664–78. doi: 10.1111/gcb.15223 (PMC7496832; doi:10.1111/gcb.15223)
Supplement: Supplementary file 3 [file GCB-26-4664-s002.docx]

Table S1. Minimum and maximum values of time of emergence for each climate driver.

**Pathway**	**Area**	**Zone**	**Time of Emergence**							
			POC Flux		O_2		pH		Temperature	
			Minimum	Maximum	Minimum	Maximum	Minimum	Maximum	Minimum	Maximu[illegible]
RCP 8.5	CCZ	PMNExpl	2028	2095	2018	2047	2023	2052	2027	[illegible]
		PMNReserve	2039	2087	2021	2055	2021	2051	2028	[illegible]
		APEI	2021	2098	2020	2055	2026	2062	2023	[illegible]
		Background	2017	>2100	2018	2059	2023	2065	2022	[illegible]
	MAR	PMSExpl	2034	2084	2032	2067	2029	2050	2026	[illegible]
		Ridge	2017	2094	2022	2073	2024	2057	2025	[illegible]
		Background	2017	2094	2022	2073	2024	2057	2025	[illegible]
		Buffer	2017	>2100	2021	2072	2019	2055	2021	[illegible]
RCP 2.6	CCZ	PMNExpl	2048	>2100	2018	2049	2021	2057	2024	[illegible]
		PMNReserve	2045	>2100	2018	2048	2018	2056	2025	[illegible]
		APEI	2021	>2100	2019	2059	2022	2061	2022	[illegible]
		Background	2017	>2100	2017	2064	2018	2068	2022	[illegible]
	MAR	PMSExpl	2035	2057	2033	2072	2029	2049	2037	[illegible]
		Ridge	2022	2087	2026	2073	2024	2055	2029	[illegible]

RCP 8.5	CCZ	PMNExpl	-2.3	-0.1	-23	-4
		PMNReserve	-2.1	0.03	-32	-5.5
		APEI	-1.8	0.48	-24	-4.7
		Background	-2.4	0.49	-34	-1.9
	MAR	PMSExpl	-3.9	-0.8	-9.4	-5.8
		Ridge	-5.2	0.45	-16	-1
		Background	-5.2	0.45	-16	-1
		Buffer	-5.6	0.47	-21	-1.2
RCP 2.6	CCZ	PMNExpl	-0.9	-0.1	-24	-5.2
		PMNReserve	-0.9	-0.1	-33	-5.7
		APEI	-0.8	0.33	-24	-4.7
		Background	-0.9	0.36	-36	-2.3
	MAR	PMSExpl	-1.9	-0.5	-7.4	-5.7
		Ridge	-2.2	0.54	-9.8	1
		Background	-2.2	0.54	-9.8	1
		Buffer	-2.4	0.73	-18	1.09

Table S3. Minimum and maximum values of climate changes for each climate driver.

**Pathway**	**Area**	**Zone**	**2041-2060**			
			POC Flux		O2	
			Minimum	Maximum	Minimum	Maximum
RCP 8.5	CCZ	PMNExpl	-0.4	0.1	-5.1	[illegible]
		PMNReserve	-0.3	0.12	-5.1	[illegible]
		APEI	-0.3	0.15	-4.5	[illegible]
		Background	-0.4	0.15	-5.1	[illegible]
	MAR	PMSExpl	-1.4	0	-5.5	[illegible]
		Ridge	-2.1	0.35	-17	[illegible]
		Background	-2.1	0.35	-17	[illegible]
		Buffer	-2.1	0.56	-23	[illegible]
RCP 2.6	CCZ	PMNExpl	-0.2	0.02	-4.7	[illegible]
		PMNReserve	-0.2	0.03	-4.7	[illegible]
		APEI	-0.2	0.03	-4.2	[illegible]
		Background	-0.3	0.05	-4.6	[illegible]
	MAR	PMSExpl	-0.8	-0.1	-4.9	[illegible]
		Ridge	-1.6	0.46	-14	[illegible]
		Background	-1.6	0.46	-14	[illegible]
		Buffer	-1.6	0.56	-18	[illegible]
			**2081-2100**			
			POC Flux		O2	
			Minimum	Maximum	Minimum	Maximum
RCP 8.5	CCZ	PMNExpl	-0.6	0.03	-12	[illegible]
		PMNReserve	-0.5	0.07	-12	[illegible]
		APEI	-0.5	0.13	-12	[illegible]
		Background	-0.8	0.12	-12	
